# What Are the Prognostic Factors for Radiographic Progression of Knee Osteoarthritis? A Meta-analysis

**DOI:** 10.1007/s11999-015-4349-z

**Published:** 2015-05-21

**Authors:** Alex N. Bastick, Janneke N. Belo, Jos Runhaar, Sita M. A. Bierma-Zeinstra

**Affiliations:** Department of General Practice, Erasmus MC, University Medical Center Rotterdam, Room NA-1923, PO Box 2040, 3000 CA Rotterdam, The Netherlands; Department of Public Health and Primary Care, Leiden University Medical Center, Leiden, The Netherlands

## Abstract

**Background:**

A previous systematic review on prognostic factors for knee osteoarthritis (OA) progression showed associations for generalized OA and hyaluronic acid levels. Knee pain, radiographic severity, sex, quadriceps strength, knee injury, and regular sport activities were not associated. It has been a decade since the literature search of that review and many studies have been performed since then investigating prognostic factors for radiographic knee OA progression.

**Questions/purposes:**

The purpose of this study is to provide an updated systematic review of available evidence regarding prognostic factors for radiographic knee OA progression.

**Methods:**

We searched for observational studies in MEDLINE and EMBASE. Key words were: knee, osteoarthritis (or arthritis, or arthrosis, or degenerative joint disease), progression (or prognosis, or precipitate, or predictive), and case-control (or cohort, or longitudinal, or follow-up). Studies fulfilling the inclusion criteria were assessed for methodologic quality according to established criteria for reviews on prognostic factors in musculoskeletal disorders. Data were extracted and results were pooled if possible or summarized according to a best-evidence synthesis. A total of 1912 additional articles were identified; 43 met our inclusion criteria. The previous review contained 36 articles, thus providing a new total of 79 articles. Seventy-two of the included articles were scored high quality, the remaining seven were low quality.

**Results:**

The pooled odds ratio (OR) of two determinants showed associations with knee OA progression: baseline knee pain (OR, 2.38 [95% CI, 1.74–3.27) and Heberden nodes (OR, 2.66 [95% CI, 1.46–8.84]). Our best-evidence synthesis showed strong evidence that varus alignment, serum hyaluronic acid, and tumor necrosis factor-α are associated with knee OA progression. There is strong evidence that sex, former knee injury, quadriceps strength, smoking, running, and regular performance of sports are not associated with knee OA progression. Evidence for the majority of determined associations, however, was limited, conflicting, or inconclusive.

**Conclusions:**

Baseline knee pain, presence of Heberden nodes, varus alignment, and high levels of serum markers hyaluronic acid and tumor necrosis factor-α predict knee OA progression. Sex, knee injury, and quadriceps strength, among others, did not predict knee OA progression. Large variation remains in definitions of knee OA and knee OA progression. Clinical studies should use more consistent definitions of these factors to facilitate data pooling by future meta-analyses.

**Electronic supplementary material:**

The online version of this article (doi:10.1007/s11999-015-4349-z) contains supplementary material, which is available to authorized users.

## Introduction

The prevalence of osteoarthritis of the knee (OA) is increasing worldwide and this burden will continue to increase owing to aging of the general population [95]. Consequent to an increase in incidence is the rise in the number of patients with knee OA who are prone to further deterioration of the knee. It therefore is important to better understand, control, and attempt to prevent further progression of disease in patients with knee OA.

In 2007, Belo et al. [4] published the first systematic review on prognostic factors for progression of knee OA. They found that generalized OA and hyaluronic acid levels were associated with progression of knee OA. Knee pain, baseline radiographic severity, sex, quadriceps strength, knee injury, and regular sport activities were not associated. For the remaining factors the evidence was limited or conflicting. Their literature search had been performed up to December 2003; however, many articles studying radiographic progression of knee OA have been published in the decade since that review. Therefore, we performed an update of the systematic review of observational studies by Belo et al. [4] to determine the currently available evidence on prognostic factors for radiographic progression of knee OA.

## Search Strategy and Criteria

### Literature Search

In the review by Belo et al. [4], the search of the literature had been performed in MEDLINE and EMBASE for all available observational studies up to December 2003. We searched in MEDLINE and EMBASE from December 2003 up to February 2013. Key words were: knee, osteoarthritis (or arthritis, or arthrosis, or degenerative joint disease), progression (or prognosis, or precipitate, or predictive), and case-control (or cohort, or longitudinal, or follow-up). Articles were reviewed for inclusion independently by two authors (ANB and JNB or JR). The following inclusion criteria were used: 85% or more of participants in the analyses for OA progression had radiographic evidence of knee OA at baseline; the study investigated determinants associated with radiographic knee OA progression; radiographic progression was the outcome measure; the study had a case-control or cohort design with a minimal 1-year followup; full text of the article was available; the study was in English, Dutch, German, or French. Studies that observed the incidence of knee OA were excluded. A detailed description of our search strategy is available online (Appendix 1. Supplemental materials are available with the online version of CORR^®^). All articles were reviewed for inclusion independently by two authors (ANB and JNB or JR). Studies that used MRI features to define OA progression were excluded. However, studies determining MRI features as prognostic factors were included.

### Methodologic Quality

The same methodologic quality assessment criteria as in the original review by Belo et al. [4] were used for this review (Table 1). These criteria were based on established criteria used in systematic reviews of prognostic factors for patients with musculoskeletal disorders and were described by Lievense et al. [49], Scholten-Peeters et al. [69], and Altman [1]. The criteria cover the internal validity and the informativeness of the study. All included articles were scored independently by two authors (ANB and JNB or JR). Cohen’s kappa coefficient (κ) was calculated to indicate the interrater agreement.

**Table 1:** Methodologic quality assessment criteria

Study population
Description of source population
Valid inclusion criteria
Sufficient description of inclusion criteria
Followup
Followup at least 1 year
Prospective or retrospective data collection
Loss to followup ≤ 20%
Information about loss to followup (selective for age, sex, or severity)
Exposure
Exposure assessment blinded for the outcome
Exposure measured identically in the studied population at baseline and followup
Outcome
Outcome assessment blinded for exposure
Outcome measured identically in the studied population at baseline and followup
Analysis
Measure of association or measures of variance given
Adjusted for age, sex, and severity

Reprinted with permission of John Wiley and Sons from Belo JN, Berger MY, Reijman M, Koes BW, Bierma-Zeinstra SM. Prognostic factors of progression of osteoarthritis of the knee: a systematic review of observational studies. *Arthritis Rheum.* 2007;57:13–26.

### Data Extraction

Study population characteristics, observed risk factors, definitions of knee OA progression, and measures of association were extracted.

### Evidence Synthesis

Odds ratios (ORs), relative risks (RRs), or hazard ratios (HRs) were pooled when there was consistency in definition of study population, measured determinants, and assessed outcome (using Review Manager [RevMan], Version 5.3; Copenhagen, Denmark: The Nordic Cochrane Centre, The Cochrane Collaboration, 2014). We tested for heterogeneity with the chi-square and I-square tests. If heterogeneity was absent, a fixed effects model was applied to calculate pooled OR through the Mantel Haenszel test. In the absence of consistency among definitions for OA, a best-evidence synthesis was used to summarize the data. The level of evidence was based on the updated guidelines by Furlan et al. [34] and was divided into the following levels: (A) strong, ie, consistent (> 75%) findings among two or more high-quality studies; (B) moderate, ie, findings in one high-quality study and consistent findings in two or more low-quality studies; (C) limited, ie, findings in one high-quality study or consistent findings in three or more low-quality studies; and (D) conflicting or inconclusive evidence, ie, less than 75% of the studies reported consistent findings, or the results were based on only one study. High quality was defined as a quality score of 9 or greater (> 65% of the maximal attainable score). When performing the best-evidence synthesis, we only differentiated between high- and low-quality studies.

### Studies Included

Of the 1912 articles identified using our search strategy, 43 met the inclusion criteria [2, 5, 7, 11, 13, 19, 20, 25–28, 30, 35, 38–44, 46, 48, 50–52, 55, 57–62, 64–66, 73, 74, 78, 85, 88, 91–93]. Belo et al. reviewed 36 articles [3, 8, 12, 14–16, 18, 21–24, 29, 31, 32, 37, 45, 47, 53, 54, 56, 63, 70–72, 75–77, 79–83, 87, 89, 94, 96]; therefore the total number of included studies was 79, studying 59 different determinants for the progression of knee OA (Table 2). Three reviewers scored 559 items for the methodologic quality assessment of the 43 newly included articles and agreed on 519 items (93%; κ = 0.79). The 53 disagreements were resolved in a single consensus meeting. Seventy-two of the 79 included articles were scored as high quality (score, 9–13), and only one article had the maximum attainable score. The remaining seven were scored as low quality, however no article was scored less than 6. Six different criteria were used for the inclusion of participants with OA and 13 definitions were applied to define radiographic OA progression. Furthermore, there were differences in how the determinants under study were measured, ie, continuous, dichotomous, or categorical with varying cut-off points.

**Table 2:** Study characteristics of the reviewed manuscripts (n = 79)

Study	Number of participants	Followup (months)	Definition of OA for inclusion	Mean age in years ± SD	Women (%)	Quality score
Sharma et al. [78], 2010	950	30	K/L	63.6 ± 7.8	62	13
Brouwer et al. [13], 2007	169	72	K/L	66.4 ± 6.7	59	12
Cerejo et al. [16], 2002	230	18	K/L	64 ± 10.8	73	12
Dieppe et al. [21], 1997	415	37.6*	K/L	65.3	68	12
Felson et al. [29], 2003	223	15 and 30	OARSI	66.2 ± 9.4	42	12
Madan-Sharma et al. [50], 2008	186	24	ACR criteria	60.2	81	12
McAlindon et al. [53], 1996	556	120	K/L	70.3	63	12
Sharma et al. [79], 2001	230	18	K/L, JSW	64.0 ± 11.1	75	12
Spector et al. [81], 1994	58	24	K/L	56.8 ± 5.9	100	12
Vilim et al. [87], 2002	48	36	K/L, JSW	62.8 (48–74)	71	12
Bagge et al. [3], 1992	74	48	K/L	NR	57	11
Benichou et al. [5], 2010	67	12	OARSI	60 ± 9	64	11
Botha-Scheepers et al. [11], 2008	86	24	ACR criteria	61	80	11
Brandt et al. [12], 1999	82	31.5*	K/L	70.1	70	11
Denoble et al. [20], 2011	69	36	K/L	64.5 ± 10.1	71	11
Dieppe et al. [23], 1993	60	60	cOA and rOA	62.2 ± 1.5	65	11
Dieppe et al. [22], 2000	349	96	K/L	65.3	68	11
Ledingham et al. [47], 1995	188	24	K/L	71 (34–91)	63	11
Miyazaki et al. [56], 2002	74	72	K/L, JSW	69.9 ± 7.8	81	11
Nevitt et al. [59], 2010	1754	30	K/L	63 ± 8	63	11
Niu et al. [61], 2009	2623	30	K/L	62.4 ± 8.0	59	11
Sharif et al. [72], 1995	75	60	K/L	64.2 ± 11.6	69	11
Sharif et al. [75], 1995	57	60	JSW	NR	NR	11
Sharif et al. [76], 2000	40	60	K/L	65.2 ± 9.9	61	11
Sharif et al. [74], 2004	115	60	K/L	63.6 ± 9.7	55	11
Sharif et al. [73], 2007	115	60	K/L	63.6 ± 9.7	55	11
Zhang et al. [96], 1998	551	96	K/L	71 (63–91)	100	11
Zhang et al. [94], 2000	473	96	K/L	71 (63–91)	100	11
Bettica et al. [8], 2002	216	48	Osteophytes, JSW	NR	100	10
Cooper et al. [18], 2000	354	61.2*	K/L	71.3	72	10
Dam et al. [19], 2009	138	21	ACR criteria	60	48	10
Doherty et al. [24], 1996	134	30	K/L	71 (41–88)	56	10
Duncan et al. [25], 2011	414	36	K/L	64.8 ± 8.1	51	10
Felson et al. [31], 1995	869	97.2*	K/L	70.8 ± 5.0	64	10
Felson et al. [30], 2007	715 + 488	30 + 120	NR^§^, ACR criteria	53 + 66	53 + 40	10
Fraenkel et al. [32], 1998	423	48	K/L	NR	67	10
Hart et al. [37], 2002	830	48	Osteophytes, JSW	54.1 ± 5.9	100	10
Kopec et al. [43], 2012	259	72	K/L	NR	65	10
Lane et al. [45], 1998	55	108	Osteophytes, JSW	66	33	10
Larsson et al. [46], 2012	74	90	OARSI	50 (32–73)	18	10
Mazzuca et al. [51], 2006	319	30	K/L	60.0 ± 9.6	84	10
McAlindon et al. [54], 1996	640	120	K/L	70.3	64	10
Miyazaki et al. [55], 2012	84	96	K/L	72.3 ± 3.1	93	10
Muraki et al. [57], 2012	1313	40	K/L	68.7 ± 11.3	75	10
Nelsonet al. [58], 2010	329	60	K/L	61.9 ± 9.7	61	10
Pavelka et al. [63], 2000	139	60	K/L	59.1 ± 8.0	76	10
Reijman et al. [66], 2007	532	72	K/L	68.6 ± 7.0	68	10
Schouten et al. [70], 1992	239	146.4*	K/L	57.2 ± 6.1	59	10
Sharma et al. [77], 2003	171	18	K/L	64.0 ± 11.1	74	10
Spector et al. [80], 1992	63	132	K/L	60 and 61	72	10
Spector et al. [82], 1997	845	48	K/L	NR	100	10
Sugiyama et al. [83], 2003	110	48	JSW	50.2 ± 6.0	100	10
Wilder et al. [88], 2009	217	67.2*	K/L	65.9 ± 9.6	61	10
Yoshimura et al. [91], 2012	1296	36	K/L	63	66	10
Zhai et al. [93], 2007	618	84	NR	56	-NR	10
Attur et al. [2], 2011	98	24	K/L	60.7	56	9
Bergink et al. [7], 2009	1248	72	K/L	66.2 ± 6.7	58	9
Bruyere et al. [14], 2003	157	36	ACR criteria	66.0 ± 7.3	76	9
Bruyere et al. [15], 2003	157	36	ACR criteria	66.0 ± 7.3	76	9
Felson et al. [27], 2005	270	30	K/L	66.6 ± 9.2	40	9
Golightly et al. [35], 2010	1583	72	K/L	60.9 ± 10.0	64	9
Harvey et al. [38], 2010	2964	30	K/L	62 ± 8	58	9
Haugen et al. [39], 2012	267	12	OARSI	61.0 ± 9.5	55	9
Kraus et al. [44], 2009	138	36	K/L	NR	74	9
Le Graverand et al. [48], 2009	141	24	K/L	56	100	9
Mazzuca et al. [52], 2004	73	30	K/L	55.2 ± 5.8	100	9
Nishimura et al. [60], 2010	92	48	K/L	71 ± 4.7	61	9
Peregoy and Wilder [64], 2011	157	72	K/L	66.5 ± 8.7	56	9
Reijman et al. [65], 2004	237	72	K/L	69.1 ± 6.9	71	9
Schouten et al. [71], 1993	239	146	K/L	57.4 ± 6.3	59	9
Wolfe and Lane [89], 2002	583	31 + 102	ACR criteria	63.4 ± 11.8	77	9
Yusuf et al. [92], 2011	155	72	K/L	59.6 ± 7.5	85	9
Fayfman et al. [26], 2009	490	120	K/L	60.5	62	8
Felson et al. [28], 2004	227	30	K/L	66.4 ± 9.4	41	8
Hunter et al. [40], 2007	595	36	Clinical symptoms	73.6 ± 2.9	60	8
Valdes et al. [85], 2004	280	120	K/L	56.9	100	8
Kerkhof et al. [41], 2010	835	72	K/L	67	64	6
Kerna et al. [42], 2009	141	36	K/L	NR	70	6
Pavelka et al. [62], 2004	89	24	ACR criteria	56.7 ± 7.2	66	6

OA = osteoarthritis; K/L = Kellgren-Lawrence score; OARSI = Osteoarthritis Research Society International atlas; ACR = American College of Rheumatology; JSW = joint space width, cOA = clinical OA; rOA = radiographic OA; NR = not reported; *mean followup in months; ^§^criteria not reported for one of the cohorts.

### Study Results

Because of the large number of studied determinants (n = 59), we pragmatically grouped our findings into five different categories: systemic factors (Table 3); disease characteristics (Table 4); intrinsic factors (Table 5); extrinsic factors (Table 6); and markers (Table 7). Some authors presented statistically significant associations to OA progression, but used p values or regression coefficients as measures of association [3, 5, 12, 14, 20, 21, 23, 31, 37, 41, 42, 44, 45, 47, 48, 52, 62, 63, 72, 74, 77, 80, 82, 85, 87, 93]. We chose to present only OR, RR, or HR as measures of associations; however, we have tabulated whether there was a significant association with OA progression in an article.

**Table 3:** Systemic factors discussed in the reviewed studies

Determinant	Study	Instrument of measurement	Definition of knee OA progression	OR/RR/HR (95% CI)	Association with OA progression*
Age (n = 3690)	Bagge et al. [3], 1992	Dichotomous	Increase K/L ≥ 1 (baseline K/L not provided)	Not provided	o
	Benichou et al. [5], 2010	< 60 versus ≥ 60 years	Change in JSW (mean difference)	Not provided	o
	Dieppe et al. [23], 1993		JSN ≥ 2 mm	Not provided	o
	Felson et al. [31], 1995		Increase K/L ≥ 1 (baseline K/L ≥ 2)	Not provided	o
	Mazzuca et al. [51], 2006	Continuous (years)	Change in JSW (mean difference)	OR 1.13 (0.87–1.48)	o
	Miyazaki et al. [56], 2002	Continuous (years)	JSN > 1 grade on a 4-grade scale	OR 1.22 (1.05–1.41)	+
	Muraki et al. [57], 2012	Per 5-year increase	Increase K/L ≥ 1 (baseline K/L ≥ 2)	OR 1.17 (1.05–1.30)	+
	Nishimura et al. [60], 2010	Continuous (years)	Increase K/L ≥ 1 (baseline K/L ≥ 2)	OR 0.93 (0.83–1.06)	o
	Schouten et al. [70], 1992	Fourth quartile versus first	Change in JSW ≥ 1 on a 9-point scale	OR 3.84 (1.10–13.4)	+
	Wolfe and Lane [89], 2002	Continuous (years)	JSN score = 3 on a 4-point scale	HR 1.00 (0.98–1.02)	o
Female sex (n = 2235)	Benichou et al. [5], 2010		Change in JSW (mean difference)	Not provided	o
	Dieppe et al. [23], 1993		JSN ≥ 2 mm	Not provided	o
	Felson et al. [31], 1995		Increase K/L ≥ 1 (baseline K/L ≥ 2)	RR 1.43 (0.80–2.58)	o
	Ledingham et al. [47], 1995		Increase K/L or JSW (cutoff not provided) Change in cyst size/number	Not provided OR 2.17 (1.13–4.15)	o +
	Miyazaki et al. [56], 2002		JSN > 1 grade on a 4-grade scale	OR 2.14 (0.34–13.5)	o
	Nishimura et al. [60], 2010		Increase K/L ≥ 1 (baseline K/L ≥ 2)	OR 1.32 (0.22–7.75)	o
	Schouten et al. [70], 1992		Change in JSW ≥ 1 on a 9-point scale	OR 0.50 (0.22–1.11)	o
	Spector et al. [80], 1992		Change JSN ≥ 1 (4-grade scale), or ≥ 10% JSW reduction	Not provided	o
	Wolfe and Lane [89], 2002		JSN score = 3 on a 4-point scale	HR 0.73 (0.44–1.19)	o
Ethnicity (n = 1091)	Kopec et al. [43], 2012	Black versus white	Increase K/L ≥ 1 (baseline K/L ≥ 2)	HR 1.67 (1.05–2.67)	+
Low bone density (n = 3057)	Hart et al. [37], 2002	Low versus high	Change JSN ≥ 1 grade on a 4-grade scale	Not provided	o
	Nevitt et al. [59], 2010	High versus low	Change JSN ≥ 0.5 grade or osteophytes ≥ 1	OR 1.3 (0.7–2.0)	o
	Zhang et al. [94], 2000	Fourth quartile (high) versus first	Increase K/L ≥ 1 (baseline K/L ≥ 2)	OR 0.1 (0.03–0.3)	−
Osteoporosis (n = 92)	Nishimura et al. [60], 2010	Present versus absent	Increase K/L ≥ 1 (baseline K/L ≥ 2)	OR 1.67 (0.44–6.28)	o
IGF-1 (n = 662)	Fraenkel et al. [32], 1998	Third tertile versus first in women	Increase K/L ≥ 1 (baseline K/L ≥ 2)	OR 0.9 (0.5–1.6)	o
		Third tertile versus first in men	Increase K/L ≥ 1 (baseline K/L ≥ 2)	OR 0.9 (0.3–3.0)	o
	Schouten et al. [71], 1993	Third tertile versus first	Change ≥ 2 on a 5-point scale for radiographic OA	OR 2.58 (1.01–6.60)	+
Metabolic syndrome (OW, HT, DL, IGT) (n = 1296)	Yoshimura et al. [91], 2012	≥ 3 components versus none	Increase K/L ≥ 1 (baseline K/L ≥ 2)	OR 2.80 (1.68–4.68)	+
		Two components versus none		OR 2.29 (1.49–3.54)	+
		One component versus none		OR 1.38 (0.91–2.08)	o
Estrogen use (n = 551)	Zhang et al. [96], 1998	Past use versus never used	Increase K/L ≥ 1 (baseline K/L ≥ 2)	OR 0.9 (0.6–1.4)	o
		Current use versus never used	Increase K/L ≥ 1 (baseline K/L ≥ 2)	OR 0.4 (0.1–1.5)	o
Uric acid concentration (n = 239)	Schouten et al. [70], 1992	High tertile versus low	Change in JSW ≥ 1 on a 9-point scale	OR 1.36 (0.46–4.02)	o
		Middle versus low	Change in JSW ≥ 1 on a 9-point scale	OR 1.05 (0.36–3.00)	o
Plasma homocysteine (n = 490)	Fayfman et al. [26], 2009	Third tertile versus first in men Third tertile versus first in women	Increase K/L ≥ 1 (baseline K/L ≥ 2)	OR 0.6 (0.1–1.1) OR 1.7 (0.8–3.8)	o o
Genetic components (n = 618)	Zhai et al. [93], 2007	Hereditability in MZ	Change ≥ 1 in JSN or osteophyte score	Not provided	o
		Hereditability in DZ		Not provided	+
SNP (n = 421)	Kerna et al. [42], 2009	rs3740199 in women	Increase JSN ≥ 1 or osteophyte grade	OR 2.66 (1.19–5.98)	+
		rs1871054	Increase JSN ≥ 1 or osteophyte grade	Not provided	o
	Valdes et al. [85], 2004	ADAM12_48	Increase K/L ≥ 1 (baseline K/L not provided)	Not provided	o
		CILP_395		Not provided	+
		TNA_106		Not provided	o
Depression/anxiety (n = 583)	Wolfe and Lane [89], 2002	Depression, yes versus no	JSN score = 3	HR 1.09 (0.93–1.28)	o
		Anxiety, yes versus no		HR 0.95 (0.84–1.08)	o

* Statistically significant association of the determinant with OA progression: + = positive association, − = negative association, o = no association (adjusted for age and sex if applicable); OA = osteoarthritis; K/L = Kellgren-Lawrence score; JSW = joint space width; JSN = joint space narrowing; IGF-1 = insulin-like growth factor 1; OW = overweight; HT = hypertension; DL = dyslipidemia; IGT = impaired glucose tolerance; MZ = monozygotic; DZ = dizygotic; SNP = single nucleotide polymorphisms; ADAM = A disintegrin and matrix metalloproteinase domain 12; CILP = cartilage intermediate-layer protein, nucleotide pyrophosphohydrolase; TNA = tetranectin (plasminogen-binding protein); OR = odds ratio; RR = relative risk; HR = hazard ratio; n = combined sample size.

**Table 4:** Disease characteristics discussed in the reviewed studies

Determinant	Study	Instrument of measurement	Definition of knee OA progression	OR/RR/HR (95% CI)	Association with OA progression*
Knee pain (n = 2444)	Cooper et al. [18], 2000	Present versus absent	Increase K/L ≥ 1 (baseline K/L ≥ 1)	OR 0.8 (0.4–1.7)	o
			Increase K/L ≥ 1 (baseline K/L ≥ 2)	OR 2.4 (0.7–8.0)	o
	Dieppe et al. [23], 1993	Present versus absent	JSN ≥ 2 mm	Not provided	o
	Miyazaki et al. [56], 2002	Present versus absent	Change JSN ≥ 1 grade on a 4-grade scale	OR 0.93 (0.78–1.11)	o
	Muraki et al. [57], 2012	Present versus absent	Increase K/L ≥ 1 (baseline K/L ≥ 2)	OR 2.63 (1.81–3.81	+
	Spector et al. [80], 1992	Present versus absent	Change JSN ≥ 1 grade on a 4-grade scale, or ≥ 10% JSN	Not provided	o
	Wolfe and Lane [89], 2002	Present versus absent	JSN score = 3 on a 4-point scale	HR 1.55 (1.07–2.24)	+
Severity Radiographic (n = 1874)	Bruyere et al. [15], 2003	Severity high versus low	JSN ≥ 0.5 mm	RR 2.39 (0.99–5.79)	o
	Duncan et al. [25], 2011	Mild PFJOA versus none^†^	Increase K/L ≥ 1 (baseline K/L ≥ 2) for TFJOA	OR 4.5 (1.8–11.2)	+
		Mild TFJOA versus none^†^	Increase K/L ≥ 1 (baseline K/L ≥ 2) for PFJOA	OR 1.7 (0.3–9.0)	o
	Ledingham et al. [47], 1995	Change ≥ 1 rOA feature versus no change	Change in attrition (cutoff not provided) Increase K/L or JSW (cutoff not provided)	OR 1.72 (1.36–2.19) Not provided	+ o
	Mazzuca et al. [51], 2006	JSW high versus low^†^	Change in JSW (mean difference)	OR 0.67 (0.49–0.91)	+
		Patellofemoral OA	Change in JSW (mean difference)	OR 3.01 (1.63–5.57)	+
	Miyazaki et al. [56], 2002	JSW, > 3 versus < 3 mm	Change JSN ≥ 1 grade on a 4-grade scale	OR 0.74 (0.25–2.19)	o
	Pavelka et al. [63], 2000	JSW (continuous)	Increase K/L ≥ 1 (baseline K/L not provided)	Not provided	o
	Wolfe and Lane [89], 2002	Initial JSN, high versus low	JSN score = 3 on a 4-point scale	HR 2.62 (2.03–3.40)	+
Clinical (n = 1317)	Dieppe et al. [21], 1997	Steinbrocker grade	JSN ≥ 2 mm, sclerosis, osteophytes	Not provided	o
	Mazzuca et al. [51], 2006	WOMAC-PF^†^	Change in JSW (mean difference)	OR 1.16 (0.92–1.47)	o
	Wolfe and Lane [89], 2002	Global severity (continuous)	JSN score = 3 on a 4-point scale	HR 1.02 (1.01–1.03)	+
		HAQ, high versus low	JSN score = 3 on a 4-point scale	HR 1.34 (0.93–1.93)	o
Heberden nodes (n = 685)	Cooper et al. [18], 2000		Increase K/L ≥ 1 (baseline K/L ≥ 1)	OR 0.7 (0.4–1.6)	o
			Increase K/L ≥ 1 (baseline K/L ≥ 2)	OR 2.0 (0.7–5.7)	o
	Nishimura et al. [60], 2010		Increase K/L ≥ 1 (baseline K/L ≥ 2)	OR 2.01 (0.60–6.76)	o
	Schouten et al. [70], 1992		Change in JSW ≥ 1 on a 9-point scale	OR 5.97 (1.54–23.1)	+
Osteoarthritis (n = 694)	Haugen et al. [39], 2012	Score hand JSN	Increase K/L ≥ 1 (baseline K/L ≥ 2)	OR 1.00 (0.93–1.08)	o
		Score hand osteophytes		OR 0.96 (0.87–1.06)	o
	Ledingham et al. [47], 1995	Multiple joints versus local joint OA	Increase K/L (cutoff not provided)	OR 2.39 (1.16–4.93)	+
			Change in attrition	OR 2.42 (1.02–5.77)	+
			Change in JSW or rOA (cutoff not provided)	Not provided	o
	Schouten et al. [70], 1992	Generalized OA	Change in JSW ≥ 1 on a 9-point scale	OR 3.28 (1.30–8.27)	+
		Localized OA	Change in JSW ≥ 1 on a 9-point scale	OR 1.17 (0.51–2.72)	o
Hand grip strength (muscle strength) (n = 1313)	Muraki et al. [57], 2012	Per 1-kg strength increase	Increase K/L ≥ 1 (baseline K/L ≥ 2)	OR 0.99 (0.96–1.01)	o
Duration of symptoms (n = 643)	Dieppe et al. [23], 1993	Continuous (years)	JSN ≥ 2 mm	Not provided	o
	Wolfe and Lane [89], 2002	Continuous (years)	JSN score = 3 on a 4-point scale	HR 1.03 (1.00–1.05)	+

* Statistically significant association of the determinant with OA progression: + = positive association, − = negative association, o = no association (adjusted for age and sex if applicable); ^†^at baseline; OA = osteoarthritis; K/L = Kellgren-Lawrence score; JSN = joint space narrowing; TFJOA = tibiofemoral joint OA; PFJOA = patellofemoral joint OA; JSW = joint space width; WOMAC-PF = physical function scale of the WOMAC; HAQ = Health Assessment Questionnaire; OR = odds ratio; RR = relative risk; HR = hazard ratio; n = combined sample size; rOA = radiographic OA.

**Table 5:** Intrinsic factors discussed in the reviewed studies

Determinant	Study	Analysis of determinant	Definition of knee OA progression	OR/RR/HR (95% CI)	Association with OA progression*
Alignment (n = 2642)	Brouwer et al. [13], 2007	Varus versus neutral	Increase K/L ≥ 1 (baseline K/L ≥ 2)	OR 2.90 (1.07–7.88)	+
		Valgus versus neutral	Increase K/L ≥ 1 (baseline K/L ≥ 2)	OR 1.39 (0.48–4.05)	o
	Cerejo et al. [16], 2002	Varus versus nonvarus (K/L 0–1)	Change JSN > 1 grade on a 4-grade scale	OR 2.50 (0.67–9.39)	+
		Varus versus nonvarus (K/L 2)		OR 4.12 (1.92–8.82)	+
		Varus versus nonvarus (K/L 3)		OR 11.0 (3.10–37.8)	+
		Valgus versus nonvalgus (K/L 2)		OR 2.46 (0.95–6.34)	o
		Valgus versus nonvalgus (K/L 3)		OR 10.4 (2.76–39.5)	+
	Hunter et al. [40], 2007	Patellar tilt, fourth versus first quartile	Medial patellofemoral change JSN ≥ 1 grade on a 4-grade scale	OR 0.19 (0.09–0.43)	−
		Sulcus angle, fourth versus first quart		OR 1.49 (0.60–3.73)	o
		Bisect offset, fourth versus first quart		OR 2.23 (1.10–4.50)	+
		Patellar tilt, fourth versus first quartile	Lateral patellofemoral change JSN ≥ 1 grade on a 4-grade scale	OR 1.13 (0.57–2.24)	o
		Sulcus angle, fourth versus first quart		OR 2.09 (0.99–4.41)	o
		Bisect offset, fourth versus first quartile		OR 0.35 (0.15–0.83)	−
	Miyazaki et al. [56], 2002	Varus versus nonvarus	Change JSN ≥ 1 grade on a 4-grade scale	OR 0.90 (0.66–1.23)	o
	Schouten et al. [70], 1992	Malaligned, present versus absent	Change JSN ≥ 1 grade on a 4-grade scale	OR 5.13 (1.14–23.1)	+
	Sharma et al. [79], 2001	Varus versus nonvarus	Change JSN ≥ 1 grade on a 4-grade scale	OR 4.09 (2.20–7.62)	+
		Varus versus mild valgus		OR 2.98 (1.51–5.89)	+
		Valgus versus nonvalgus		OR 4.89 (2.13–11.2)	+
		Valgus versus mild varus		OR 3.42 (1.31–8.96)	+
	Sharma et al. [78], 2010	Valgus versus neutral	Change medial JSN ≥ 1 grade on a 4-grade scale	OR 0.34 (0.21–0.55)	−
		Varus versus neutral		OR 3.59 (2.62–4.92)	+
		Valgus versus neutral	Change lateral JSN ≥ 1 grade on a 4-grade scale	OR 4.85 (3.17–7.42)	+
		Varus versus neutral		OR 0.12 (0.07–0.21)	−
	Yusuf et al. [92], 2011	Varus (< 182°) versus nonvarus	Change JSN ≥ 1 grade on a 6-grade scale	RR 2.3 (1.4–3.1)	+
		Valgus (> 184°) versus nonvalgus		RR 1.7 (0.97–2.6)	o
		Malaligned, BMI > 25 kg/m^2^		RR 4.1 (1.8–6.1)	+
Adduction moment (n = 74)	Miyazaki et al. [56], 2002	≥ 5 versus < 5 (% weight x height)	Change JSN ≥ 1 grade on a 4-grade scale	OR 6.46 (2.40–17.5)	+
Knee injury (n = 207)	Cooper et al. [18], 2000	Yes versus no	Increase K/L ≥ 1 (baseline K/L ≥ 1)	OR 1.2 (0.5–3.0)	o
			Increase K/L ≥ 1 (baseline K/L ≥ 2)	OR 1.1 (0.3–4.4)	o
	Schouten et al. [70], 1992	Knee injury: yes versus no	Change JSN ≥ 1 grade on a 4-grade scale	OR 2.62 (0.93–7.36)	o
		Sport injury: yes versus no	Change JSN ≥ 1 grade on a 4-grade scale	OR 0.62 (0.17–2.19)	o
Bone marrow lesions/edema (n = 186)	Madan-Sharma et al. [50], 2008	Present versus absent	JSN > 1 grade on a 4-grade scale	RR 0.9 (0.18–3.0)	o
Subchondral bone cysts (MRI) (n = 186)	Madan-Sharma et al. [50], 2008	Present versus absent	JSN > 1 grade on a 4-grade scale	RR 1.6 (0.5–4.0)	o
Cartilage loss (MRI) (n = 186)	Madan-Sharma et al. [50], 2008	Present versus absent	JSN > 1 grade on a 4-grade scale	RR 3.0 (0.5–9.6)	o
Joint effusion (n = 186)	Madan-Sharma [50], 2008	Present on MRI	JSN > 1 grade on a 4-grade scale	RR 0.6 (0.6–1.8)	o
Meniscal damage (n = 186)	Madan-Sharma et al. [50], 2008	Present versus absent on MRI	JSN > 1 grade on a 4-grade scale	RR 8.91 (1.1–22.8)	+
Meniscectomy (n = 239)	Schouten et al. [70], 1992	Yes versus no	Change JSN ≥ 1 grade on a 4-grade scale	OR 2.28 (0.57–9.03)	o
Chondrocalcinosis (n = 239)	Schouten et al. [70], 1992	Yes versus no	Change JSN ≥ 1 grade on a 4-grade scale	OR 2.01 (0.55–7.42)	o
Osteophytes tibiofemoral (n = 337)	Benichou et al. [5], 2010	Definite versus not	Change in JSW (mean difference)	Not provided	o
	Felson et al. [27], 2005	Ipsilateral score Contralateral score	Change JSN ≥ 1 grade on a 4-grade scale	OR 1.9 (1.5–2.5)	+
				OR 0.6 (0.5–0.8)	−
Knee ROM (n = 92)	Nishimura et al. [60], 2010	Mean ROM	Increase K/L ≥ 1 (baseline K/L ≥ 2)	OR 0.94 (0.89–0.99)	−

* Statistically significant association of the determinant with OA progression: + = positive association, − = negative association, o = no association (adjusted for age and sex if applicable); OA = osteoarthritis; K/L = Kellgren-Lawrence score; JSN = joint space narrowing; JSW = joint space width; OR = odds ratio; RR = relative risk; HR = hazard ratio; n = combined sample size.

**Table 6:** Extrinsic factors discussed in the reviewed studies

Determinant	Study	Analysis of determinant	Definition of knee OA progression	OR/RR/HR (95% CI)	Association with OA progression*
BMI (n = 6791)	Benichou et al. [5], 2010	< 30 versus ≥ 30 kg/m^2^	Change in JSW (mean difference)	Not provided	+
	Cooper et al. [18], 2000	Highest tertile versus lowest	Increase K/L ≥ 1 (baseline K/L ≥ 1)	OR 2.6 (1.0–6.8)	+
			Increase K/L ≥ 1 (baseline K/L ≥ 2)	OR 1.3 (0.3–5.0)	o
	Dieppe et al. [23], 1993	Continuous	JSN ≥ 2 mm or knee surgery	Not provided	o
	Felson et al. [28], 2004	Per 2-unit increase (§)	Change JSN ≥ 1 grade on a 4-grade scale	OR 0.98 (0.8–1.4)	o
		As §, with 3°–6° malalignment		OR 1.23 (1.0–1.4)	+
		As §, with ≥ 7° malalignment		OR 0.93 (0.7–1.2)	o
	Ledingham et al. [47], 1995	Continuous	Change in JSW (cutoff not provided)	OR 1.07(1.02–1.14)	+
			Change in osteophytes (cutoff not provided)	OR 1.06 (1.00–1.12)	+
			Change in K/L (cutoff not provided)	Not provided	o
	LeGraverand et al. [48], 2009	< 30 versus ≥ 30 kg/m^2^	Change in JSW (mean difference)	Not provided	o
	Miyazaki et al. [56], 2002	Continuous	JSN ≥ 1 grade on a 4-grade scale	OR 1.21 (0.91–1.61)	o
	Muraki et al. [57], 2012	Per 5-unit increase	Increase K/L ≥ 1 (baseline K/L ≥ 2)	OR 1.43 (1.16–1.77)	+
	Nishimura et al. [60], 2010	Continuous	Increase K/L ≥ 1 (baseline K/L ≥ 2)	OR 0.93 (0.78–1.11)	o
	Niu et al. [61], 2009	< 25 versus ≥ 30 kg/m^2^	Increase JSN ≥ 0.5 grade	RR 1.1 (0.9–1.4)	o
	Reijman et al. [66], 2007	≤ 25 versus > 27.5 kg/m^2^	Increase JSN ≥ 1 mm	OR 1.4 (0.8–2.6)	o
			Increase K/L ≥ 1 (baseline K/L ≥ 2)	OR 2.1 (1.2–3.7)	+
	Schouten et al. [70], 1992	Second quartile versus first	Change in JSW ≥ 1 on a 9-point scale	OR 1.77 (0.48–6.50)	o
		Third quartile versus first		OR 5.28 (1.54–18.1)	+
		Fourth quartile versus first		OR 11.1 (3.28–37.3)	+
	Spector et al. [81], 1994	Third tertile versus first	Increase K/L or JSN (cutoff not provided)	RR 4.69 (0.63–34.8)	o
	Wolfe and Lane [89], 2002	Continuous	JSN score = 3	HR 1.03 (1.00–1.06)	+
	Yusuf et al. [92], 2011	BMI 25–30 versus < 25	Change JSN ≥ 1 grade on a 6-grade scale	RR 2.4 (1.3–3.6)	+
		BMI >30 versus < 25	Change JSN ≥ 1 grade on a 6-grade scale	RR 2.9 (1.7–4.1)	+
Quadriceps strength (n = 253)	Brandt et al. [12], 1999	Progressive versus nonprogressive group^†^	Increase K/L ≥ 1 (baseline K/L not provided)	Not provided	o
	Sharma et al. [77], 2003	High versus low strength^†^	Increase JSN ≥ 1	Not provided	o
Leg length inequality (n = 4547)	Golightly et al. [35], 2010	Leg length inequality versus no inequality	Increase K/L ≥ 1 (baseline K/L ≥ 1)	HR 1.22 (0.82–1.80)	o
			Increase K/L ≥ 1 (baseline K/L ≥ 2)	HR 1.83 (1.10–3.05)	+
	Harvey et al. [38], 2010	≥ 1 cm versus no inequality, shorter leg	JSN ≥ 1 grade or knee surgery	OR 1.3 (1.0–1.7)	+
		≥ 2 cm versus no inequality, shorter leg		OR 1.4 (0.5–3.7)	o
AP knee laxity (n = 84)	Miyazaki et al. [55], 2012	Before exercise	Increase K/L ≥ 1 (baseline K/L ≥ 1) or radiographic cartilage loss > 0.2 mm annually	OR 1.29 (0.54–3.08)	o
		Enhanced laxity resulting from exercise		OR 4.15 (1.12–15.4)	+
Running (n = 294)	Lane et al. [45], 1998	Dichotomous^‡^	Increase ≥ 1 on JSW and osteophyte score	Not provided	o
	Schouten et al. [70], 1992	Dichotomous^†^	Change in JSW ≥ 1 on a 9-point scale	OR 0.53 (0.17–1.68)	o
Regular sports (n = 593)	Cooper et al. [18], 2000	Dichotomous^†^	Increase K/L ≥ 1 (baseline K/L ≥ 1)	OR 0.7 (0.4–1.6)	o
			Increase K/L ≥ 1 (baseline K/L ≥ 2)	OR 0.9 (0.3–2.5)	o
	Schouten et al. [70], 1992	Physical activity^‡^	Change in JSW ≥ 1 on a 9-point scale	OR 0.43 (0.11–1.76)	o
		Walking^‡^		OR 1.47 (0.36–6.03)	o
		Standing (medium versus low)^‡^		OR 3.80 (1.03–14.0)	+
		Standing (high versus low)^‡^		OR 2.09 (0.43–10.3)	o
Nutritional variables (n = 3381)	Bergink et al. [7], 2009	Vitamin D intake (low versus high)	Increase K/L ≥ 1 (baseline K/L ≥ 2)	OR 7.7 (1.3–43.5)	−
		Serum vitamin D (low versus high)	Increase K/L ≥ 1 (baseline K/L ≥ 2)	OR 2.1 (0.6–7.4)	o
	Felson et al. [30], 2007	Vitamin D serum levels < 20 ng/mL	Change JSN ≥ 1 grade on a 4-grade scale, Framingham	OR 0.83 (0.54–1.27)	o
		Vitamin D serum levels < 20 ng/mL	Change JSN ≥ 1 grade on a 4-grade scale, BOKS study	OR 0.63 (0.35–1.14)	o
	McAlindon et al. [53], 1996	Vitamin D intake (middle versus high)	Increase JSN ≥ 1	OR 2.99 (1.06–8.49)	−
		Serum vitamin D (middle versus high)	Increase JSN ≥ 1	OR 2.83 (1.02–7.85)	−
	McAlindon et al. [54], 1996	Vitamin C intake (middle versus low)	Increase K/L ≥ 1	OR 0.32 (0.14–0.77)	−
		β-carotene intake (high versus low)		OR 0.42 (0.19–0.94)	−
		Vitamin E (high versus low)		OR 0.68 (0.28–1.64)	o
	Peregoy and Wilder [64], 2011	Vitamin C intake	Increase K/L ≥ 1 (baseline K/L ≥ 2)	RR 0.94 (0.79–1.12)	o
	Wilder et al. [88], 2009	Vitamin intake in general	Increase K/L ≥ 1 (baseline K/L ≥ 2)	RR 0.93 (0.87–0.99)	−
Smoking (n = 331)	Nishimura et al. [60], 2010	Yes versus no	Increase K/L ≥ 1 (baseline K/L ≥ 2)	OR 0.73 (0.09–6.15)	o
	Schouten et al. [70], 1992	Past smoker versus never	Change in JSW ≥ 1 on a 9-point scale	OR 1.07 (0.38–3.04)	o
		Current smoker versus never	Change in JSW ≥ 1 on a 9-point scale	OR 0.96 (0.34–2.75)	o

* Statistically significant association of the determinant with OA progression: + = positive association, − = negative association, o = 1o association (adjusted for age and sex if applicable); ^†^assessed at baseline; ^‡^assessed at followup; OA = osteoarthritis; JSW = joint space width; K/L = Kellgren-Lawrence score; JSN = joint space narrowing; OR = odds ratio; RR = relative risk; HR = hazard ratio; n = combined sample size.

**Table 7:** Markers discussed in the reviewed studies

Marker	Study	Instrument of measurement	Definition of knee OA progression	OR/RR/HR (95% CI)	Association with OA progression*
CRP (serum) (n = 1720)	Kerkhof et al. [41], 2010	Continuous	Increase K/L ≥ 1 (baseline K/L ≥ 2) or surgery	Not provided	o
	Sharif et al. [76], 2000	Continuous	JSN ≥ 2 mm or knee surgery	OR 1.12 (0.81–1.55)	o
	Spector et al. [82], 1997	Continuous	Increase K/L ≥ 1 (baseline K/L not provided)	Not provided	+
IL-1β (serum) (n = 184)	Attur et al. [2], 2011	Increased versus normal	Increase K/L ≥ 1 or > 30% JSW reduction	OR 3.2 (1.2–8.7)	+
	Botha-Scheepers et al. [11], 2008	Fourth quartile versus first	Change JSN ≥ 1 grade on a 4-grade scale	RR 1.3 (0.5–2.0)	o
IL-10 (serum) (n = 86)	Botha-Scheepers et al. [11], 2008	Fourth quartile versus first	Change JSN ≥ 1 grade on a 4-grade scale	RR 4.3 (1.7–6.2)	+
IL-1Ra (serum) (n = 86)	Botha-Scheepers et al. [11], 2008	Fourth quartile versus first	Change JSN ≥ 1 grade on a 4-grade scale	RR 2.1 (0.7–3.9)	o
TNFα (serum) (n = 253)	Attur et al. [2], 2011	Increased versus normal	Increase K/L ≥ 1 or > 30% JSW reduction	OR 8.9 (2.6–30.8)	+
	Botha-Scheepers et al. [11], 2008	Fourth quartile versus first	Change JSN ≥ 1 grade on a 4-grade scale	RR 6.1 (1.4–9.8)	+
	Denoble et al. [20], 2011	Continuous	Change in osteophyte score	Not provided	+
TGF-β1 (serum) (n = 329)	Nelson et al. [58], 2010	Continuous	Increase K/L ≥ 1 (baseline K/L ≥ 1)	HR 1.04 (0.41–2.65)	o
			Increase K/L ≥ 1 (baseline K/L ≥ 2)	HR 1.10 (0.46–2.63)	o
Hyaluronic acid (serum) (n = 361)	Bruyere et al. [14], 2003	High level versus low	Change in mean JSW (cutoff not provided)	Not provided	+
	Pavelka et al. [62], 2004	High level versus low	Change in mean JSW (cutoff not provided)	Not provided	+
	Sharif et al. [72], 1995	High level versus low	JSN ≥ 2 mm or knee surgery	Not provided	+
	Sharif et al. [76], 2000	High level versus low	JSN ≥ 2 mm or knee surgery	OR 2.32 (1.16–4.66)	+
Keratan sulfate (serum) (n = 232)	Bruyere et al. [14], 2003	High level versus low	Change in mean JSW (cutoff not provided)	Not provided	+
	Sharif et al. [72], 1995	High level versus low	JSN ≥ 2 mm or knee surgery	Not provided	o
COMP (serum) (n = 466)	Bruyere et al. [14], 2003	High level versus low	Change in mean JSW (cutoff not provided)	Not provided	o
	Pavelka et al. [62], 2004	High level versus low	Change in mean JSW (cutoff not provided)	Not provided	o
	Sharif et al. [75], 1995	High level versus low	JSN ≥ 2 mm or knee surgery	Not provided	+
	Sharif et al. [74], 2004	OA progression versus nonprogession	JSN ≥ 2 mm or knee surgery	Not provided	+
	Vilim et al. [87], 2002	High level versus low	JSN > 0.5 mm	Not provided	+
Pentosidine (serum) (n = 89)	Pavelka et al. [62], 2004	High level versus low	Change in mean JSW (cutoff not provided)	Not provided	+
YKL-40 (serum) (n = 89)	Pavelka et al. [62], 2004	High level versus low	Change in mean JSW (cutoff not provided)	Not provided	o
MMP-9 (serum) (n = 89)	Pavelka et al. [62], 2004	High level versus low	Change in mean JSW (cutoff not provided)	Not provided	o
TIMP-9 (serum) (n = 89)	Pavelka et al. [62], 2004	High level versus low	Change in mean JSW (cutoff not provided)	Not provided	o
PIIANP (serum) (n = 115)	Sharif et al. [73], 2007	Fourth quartile versus first	JSN ≥ 2 mm or knee surgery	RR 3.2 (1.1–9.0)	+
CTX-II (urine) (n = 490)	Dam et al. [19], 2009	Third tertile versus first	Increase K/L ≥ 1 (disregarding baseline K/L)	OR 2.3	o
		Third tertile versus first	JSN > mean JSN of non-OA control group (K/L ≤ 1)	OR 1.8	o
	Reijman et al. [65], 2004	Fourth quartile versus first	JSN ≥ 2 mm	OR 6.0 (1.2–30.8)	+
		Fourth quartile versus first	JSN ≥ 1.5 mm	OR 1.8 (0.8–4.1)	o
		Fourth quartile versus first	JSN ≥ 1 mm	OR 1.1 (0.7–1.7)	o
	Sharif et al. [73], 2007	> median versus ≤ median	JSN ≥ 2 mm or knee surgery	RR 3.4 (1.2–9.4)	+
ARGS (synovial) (n = 74)	Larsson et al. [46], 2012	Baseline level ARGS > followup level ARGS	≥ 1-unit increase OARSI score	OR 6.77 (1.38–33.2)	+
IL-18 (synovial) (n = 69)	Denoble et al. [20], 2011	Continuous	Change in osteophyte score	Not provided	+
FSA (radiographic) (n = 138)	Kraus et al. [44], 2009	FD progression versus nonprogression	Medial JSN ≥ 1 or osteophyte formation	Not provided	+
Bone scintigraphy (n = 73)	Mazzuca et al. [52], 2004	^99m^Tc-MDP uptake	Change in JSW (mean difference)	Not provided	o

* Statistically significant association of the determinant with OA progression: + = positive association, − = negative association, o = no association (adjusted for age and sex if applicable); OA = osteoarthritis; K/L = Kellgren-Lawrence score; JSN = joint space narrowing; CRP = C-reactive protein; IL = interleukin; TNF = tumor necrosis factor; YKL-40 = chitinase-3-like protein 1; JSW = joint space width; TGF = transforming growth factor; C2C = collagen type II cleavage; COMP = cartilage oligomeric matrix protein; MMP = matrix metalloproteinase; TIMP = tissue inhibitors of metalloproteinase; PIIANP = N-propeptide of type IIA collagen; CTX-II = crosslinked C-telopeptide; ARGS = aggrecan neoepitope amino acid sequence; FSA = fractal signature analysis; FD = fractal dimension (horizontal and vertical); OR = odds ratio; RR = relative risk; HR = hazard ratio; n = combined sample size.

### Sensitivity Analysis

For factors in which we were forced to use a best-evidence synthesis, we conducted a sensitivity analysis to check whether differences in sample size could have altered our conclusions. Additionally we checked whether large variances in followup could have led to different conclusions.

## Results

Summaries of the results for systemic factors, disease characteristics, intrinsic factors, extrinsic factors, and markers are available (Appendix 2. Supplemental material is available with the online version of CORR^®^.).

### Pooled Results

**Fig. 1:**
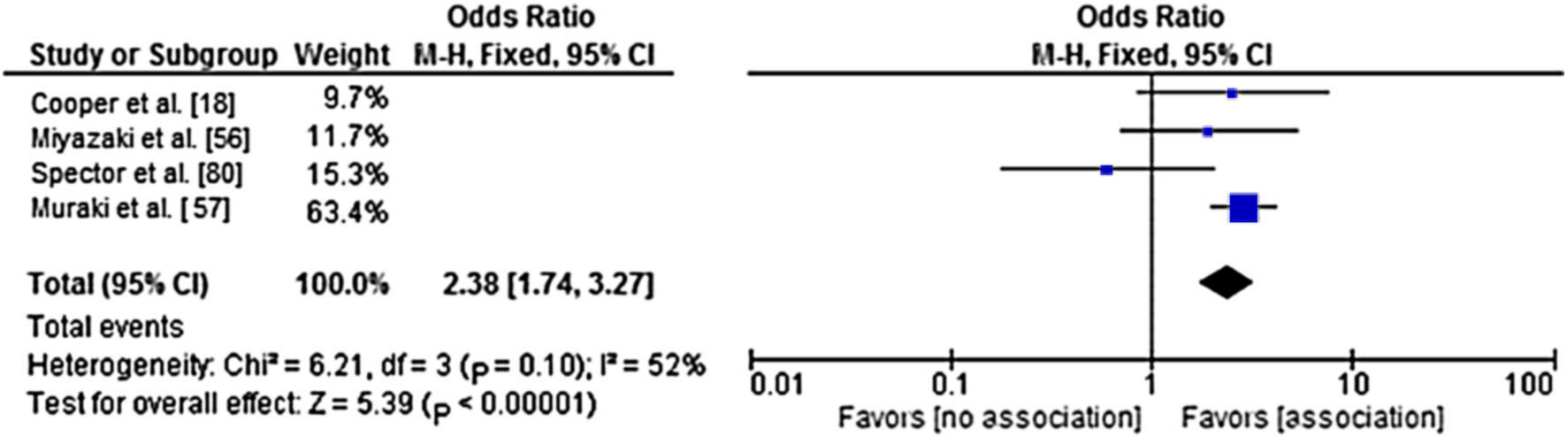
A forest plot for the pooled odds ratio (OR) shows the association between the presence of knee pain at baseline and radiographic progression of knee osteoarthritis (OA). The OR can deviate from the OR in Table 4 because pooled ORs were obtained through crude ORs, as opposed to the adjusted OR in Table 4. The results from Dieppe and Wolfe for pooling were not available and were not included in this analysis. The results from the chi-square and I^2^ tests indicate homogeneity between the studies. M–H = Mantel Haenszel test; Fixed = fixed effects model; df = degrees of freedom.

**Fig. 2:**
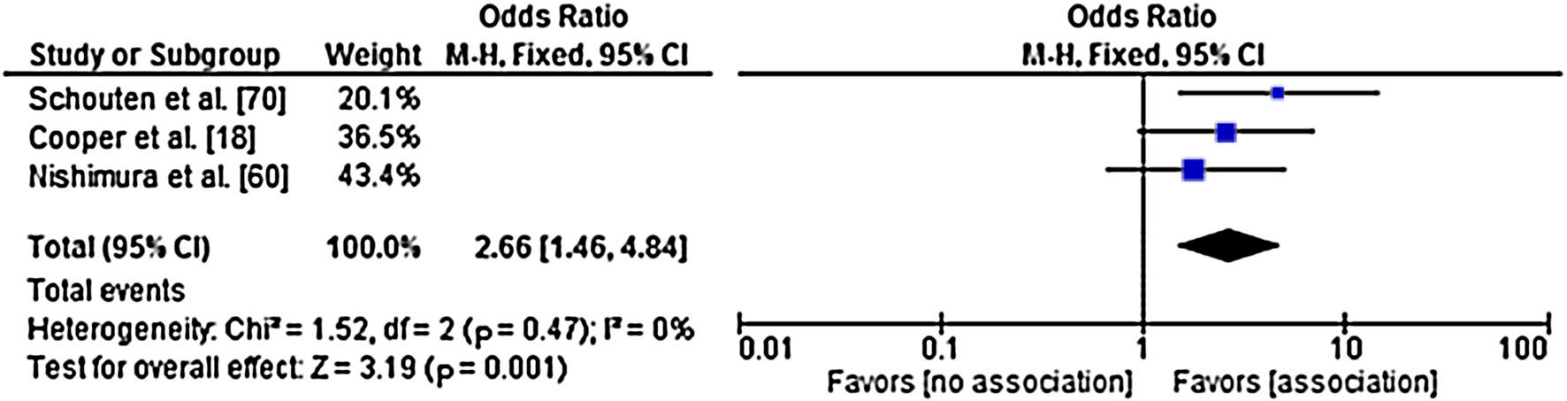
A forest plot for the pooled odds ratio (OR) shows the association between the presence of Heberden nodes at baseline and radiographic progression of knee osteoarthritis (OA). The OR can deviate from that in Table 4 because pooled ORs were obtained through crude ORs, as opposed to the adjusted OR in Table 4. The results from the chi-square and I^2^ tests indicate homogeneity between the studies. M–H = Mantel Haenszel test; Fixed = fixed effects model; df = degrees of freedom.

The presence of knee pain at baseline and Heberden nodes were associated with the progression of knee OA. The pooled ORs based on pools of studies with consistency among the definitions for OA inclusion, OA progression, and the determinant under study, were 2.38 for knee pain at baseline (95% CI,1.74–3.27; I^2^ = 52%) (Fig. 1) and 2.66 for the presence of Heberden nodes (95% CI, 1.46–8.84); I^2^ = 0%) (Fig. 2). Because of the large number of determinants with only a restricted number of studies per determinant and owing to lack of consistency between the reviewed studies regarding inclusion criteria, outcome measures, and measures of association, statistical pooling was not possible for the majority of the determinants.

### Best-evidence Synthesis

For the remaining determinants, we applied a best-evidence synthesis, which showed that based on consistent findings in multiple high-quality studies, there seems to be strong evidence that varus alignment, serum TNFα level, and serum hyaluronic acid level are associated with radiographic progression of knee OA. There also is strong evidence that sex (female), former knee injury, quadriceps strength, smoking, running, and regular performance of sports are not associated with progression of knee OA.

There was moderate evidence showing that a higher dietary intake of vitamin D is inversely associated with progression of knee OA. Thus far, there is limited evidence that ethnicity, metabolic syndrome, genetic components adduction moment, meniscal damage, knee ROM, general vitamin and β-carotene intake, serum levels IL-10 and N-propeptide of type II collagen, synovial levels aggrecan neoepitope amino acid sequence and IL-18, and fractal dimension progression on radiographic fractal signature analysis are associated with progression of knee OA. There also is limited evidence that knee OA progression is not associated with osteoporosis; past or present estrogen use; uric acid concentrations; depression or anxiety; hand grip (muscle) strength; bone marrow lesions or edema; meniscectomy; chondrocalcinosis; MRI-detected subchondral bone cysts, cartilage loss, or joint effusion; AP knee laxity; vitamin E intake; serum levels IL-1Ra and transforming growth factor-β1; and ^99m^Tc-MDP uptake on bone scintigraphy.

Conflicting evidence was found for the associations between knee OA progression and age; low bone density; serum insulin growth factor-1 level; baseline radiographic or clinical OA severity; generalized osteoarthritis; duration of symptoms; valgus alignment or malalignment in general; past knee injury; the presence of tibiofemoral osteophytes; BMI; leg length inequality; serum vitamin D level; dietary intake of vitamin C; serum C-reactive protein, IL-1β, keratan sulfate, and serum cartilage oligometric matrix protein levels, and urinary crosslinked C-telopeptide level. Inconclusive evidence was found for the determined associations between knee OA progression and the single nucleotide polymorphisms CILP_395 (cartilage intermediate-layer proteins) and rs3740199, patellofemoral alignment, and serum pentosidine levels. There also was inconclusive evidence for no associations found between knee OA progression and the single nucleotide polymorphisms rs1871054, ADAM12_48 (A disintegrin and matrix metalloproteinase domain 12), and TNA_106 (tetranectin plasminogen-binding protein), and serum levels of YKL-40 (chitinase-3-like protein 1), MMP-9 (matrix metalloproteinase-9); and TIMP-9 (tissue inhibitors of metalloproteinase).

### Sensitivity Analysis

In this analysis, we tested whether conclusions from relatively small studies (less than 200) incorrectly influenced conclusions drawn from larger studies with more statistical power studying the same determinant, or that results from studies with a relatively short followup (cutoff 24 months) altered conclusions from studies with a longer followup. Our sensitivity analysis found that our conclusions did not change across the range of clinically plausible differences in followup duration or sample size regarding the strong, moderate, or conflicting evidence we found for the various presented determinants.

## Discussion

We performed an updated systematic review of available evidence regarding prognostic factors for radiographic knee OA progression. We found that there is strong evidence that baseline knee pain and Heberden nodes, varus alignment, and high baseline serum levels of hyaluronic acid and TNFα are predictive for knee OA progression. There also seems to be strong evidence that sex (female), former knee injury, quadriceps strength, smoking, running, and regular performance of sports are not predictive for progression of knee OA. For all other studied factors in our review, the evidence is limited, conflicting, or inconclusive. In the best-evidence synthesis, we considered only significant associations as associated prognostic factors. However, several of the included articles had small sample sizes, which consequently can lead to lower statistical power and more often to failure to detect differences that might be present.

A possible limitation to our inclusion criteria was addressed by Zhang et al. [97]. They reported that, unlike randomized trials, observational studies of patients with preexisting disease are subject to various biases that may account for discrepancies found between risk factors for disease incidence and progression. They hypothesized that risk factors actually might exist for progressive knee OA but that flaws in study design and the measure of disease progression may prevent us from detecting risk factors [97]. Having cited their article, it seems reasonable that there is the possibility that we have not determined all risk factors for progression of knee OA, because some factors might not have achieved significance in multivariable analyses in a study and thus were not included in our evidence synthesis. Nonetheless, we believe we have summarized all presently known risk factors of which a possible association with knee OA progression has been studied.

We acknowledge that when applying a best-evidence synthesis, one might unjustly conclude that there may be conflicting or strong evidence for or against an association of the determinant under study with knee OA. We would have preferred to pool the data of all included studies. However, because of large variation in criteria used in the articles for defining disease, or disease progression, pooling of the data generally was not possible. We encountered six different criteria that were used for the inclusion of OA (Table 2). Another approximately 13 different definitions were applied for OA progression (Tables 3–7). Furthermore, there were differences in how the determinants under study were measured, (continuous, dichotomous, or categorical), and varying cutoff points were used. As previously described, we pooled the results for “knee pain” and “Heberden nodes” for which both results showed associations with the progression of knee OA. This is different from the conclusions we would have drawn from a best-evidence synthesis, which would show conflicting evidence for both determinants. In our opinion, it is likely that more of the conflicting associations we presented are attributable to the differences in definitions of knee OA or knee OA progression. For example, the conflicting evidence for BMI probably would be altered if statistical pooling was feasible; given that all 11 significant risk estimates (OR/RR/HR) regarding BMI were positive associations and that six of the 12 nonsignificant associations also were positive associations, it seems likely that if pooled, the combined overall association between BMI and knee OA would be a positive, significant one. In addition, the conflicting evidence for age, seven of the 10 presented analyses (70%) showed no significant association, falling just short for the criteria for ascertaining strong evidence (> 75%) for no association between age and OA progression.

In the original review by Belo et al. [4] and in a review by van Dijk et al. [86], the evidence for association between varus alignment and OA progression was limited. However, a couple studies have been performed since these reviews were published that have determined significant associations with varus alignment, which enabled us to conclude that there is strong evidence for this finding. The latter is in accordance with results published in later systematic reviews by Tanamas et al. [84] and Chapple et al. [17]. Except for the original review by Belo et al., there are to our knowledge no other reviews available that have determined the predictive value of serum hyaluronic acid levels and OA progression [9]. In addition, to our knowledge, no reviews have been published assessing the predictive value of serum level TNFα for knee OA progression.

We found strong evidence that sex was not associated with knee OA progression, as did Belo et al. [4]. This is in contrast to the earlier reviews published by van Dijk et al. [86] and Chapple et al. [17]. van Dijk et al. found limited evidence for the absence of an association with sex, but they included articles that used physical functioning as an outcome measure. Chapple et al. found conflicting evidence; however, their evidence was based on four analyses of three studies, which also are included in our review [21, 47, 70]. Three of the four analyses were consistent (no association); one was conflicting (significant association) [47]. Our evidence synthesis was based on 10 analyses, of which nine analyses were consistent (no association), consequently outweighing the one conflicting finding. van Dijk et al. and Chapple et al. reported limited evidence for the absence of an association between quadriceps strength and knee OA progression. This is consistent with our finding; however, our conclusion is based on more evidence. Consistent results also were found for regular performance of sports, in which van Dijk et al. reported limited and Chapple et al. reported strong evidence for absence of an association. However, in articles by Fransen and McConnell [33] and Bennell and Hinman [6] reviewing the effect of exercise therapy in patients with knee OA, the authors reported that exercise has a short-term benefit in patients with knee OA, although the magnitude of the reported benefit is small. This highlights the importance of the need to understand the working mechanism of exercise therapy.

A topic of considerable interest is the potential association between BMI and knee OA progression. Previous reviewers have established a positive association between BMI and incident knee OA [10, 95]. However, the evidence for an association between BMI and progression of knee OA remains conflicting in our review, which is consistent with the findings by Belo et al. [4] and Chapple et al. [17].

Noteworthy is the lack of overlap in evidence for prognostic factors for hip and knee OA progression. In two large reviews studying prognostic factors for hip OA, Lievense et al. [49] provided strong evidence for an association between hip OA progression with type of hip migration and with atrophic bone response. They also presented strong evidence for the absence of an association with BMI. Wright et al. [90] reported strong evidence for association of hip OA progression with age, joint space width at entry, femoral head migration, femoral osteophytes, bony sclerosis, baseline hip pain, and certain hip OA severity indexes. They also provided strong evidence for the absence of an association with acetabular osteophytes. The discrepancy between the findings for hip and knee OA is unclear but could be attributable to the difference in the number of studies available determining risk factors for progression of hip or knee OA [9].

Future research on the true relationship between prognostic factors for radiographic progression of knee OA is needed, mainly on the factors where conflicting evidence was presented (eg, age, baseline OA severity, BMI). Furthermore, we presented limited, inconclusive, or conflicting evidence on many factors with potential associations with OA progression. It would be important to investigate determinants that can be influenced or modified to reduce the risk of OA progression, perhaps including metabolic syndrome, bone marrow lesions, or osteoporosis. Moreover, there would be obvious advantages to testing the effect of new or existing disease-modifying pharmacologic or surgical interventions in patients with an established increased risk of OA progression.

We found strong evidence that baseline knee pain and Heberden nodes, varus alignment, and high baseline serum levels of hyaluronic acid and TNFα are predictive for knee OA progression. Sex (female), former knee injury, quadriceps strength, smoking, running, and regular performance of sports are not predictive for progression of knee OA. Many studies have been performed and are being performed determining risk factors for knee OA progression, but the variability in how OA and OA progression are defined across the relevant studies remains an impediment to pooling the available evidence. We strongly recommend future researchers use uniform definitions of determinants, disease, and disease progression; it would enable more precise determination of possible risk factors for knee OA progression through meta-analyses. The majority of the included studies used the Kellgren-Lawrence classification as definition of disease and disease progression. This classification has been criticized because the criteria have been described and interpreted differently in various studies [67]. However, the Kellgren-Lawrence criteria provide a reliable classification of knee OA and OA progression, given that the original description of the criteria are applied [67, 68]. We therefore recommend that future researchers use the Kellgren-Lawrence classification to define radiographic OA and OA progression. Furthermore, considering that some MRI scoring systems have been and currently are being developed to define knee OA progression [36], it seems preferable that the same MRI scoring system would be used universally in future studies on prognostic factors for knee OA progression. We would like to call on expert committees, such as the Osteoarthritis Research Society International (OARSI) for OA Imaging to announce their recommendations on this important topic.

## Electronic supplementary material

Below is the link to the electronic supplementary material.

Supplementary material 1 (DOC 63 kb)

Supplementary material 2 (DOC 291 kb)
