# Supplementary material for: What Are the Prognostic Factors for Radiographic Progression of Knee Osteoarthritis? A Meta-analysis
Source: Clin Orthop Relat Res. 2015 May 21;473(9):2969–89. doi: 10.1007/s11999-015-4349-z (PMC4523522; doi:10.1007/s11999-015-4349-z)
Supplement: Supplementary file 1 — Supplementary material 1 (DOC 63 kb) [file 11999_2015_4349_MOESM1_ESM.doc]

**PubMed**

(knee[tw] OR knees[tw]) AND (arthrosis[tw] OR arthrot*[tw] OR degenerative arthrit*[tw] OR degenerative joint disease*[tw] OR osteoarthrit*[tw] OR osteo-arthrit*[tw] OR osteoarthro*[tw] OR osteo-arthro*[tw]) AND (progress*[tw] OR exacerbat*[tw] OR precipit*[tw] OR prognos*[tw] OR predict*[tw]) AND (clinical trial*[tw] OR case[tw] OR follow-up*[tw] OR longitud*[tw] OR prospect*[tw] OR retrospect*[tw]) AND 0:2003[dp]

682

(knee[tw] OR knees[tw]) AND (arthrosis[tw] OR arthrot*[tw] OR degenerative arthrit*[tw] OR degenerative joint disease*[tw] OR osteoarthrit*[tw] OR osteo-arthrit*[tw] OR osteoarthro*[tw] OR osteo-arthro*[tw]) AND (progress*[tw] OR exacerbat*[tw] OR precipit*[tw] OR prognos*[tw] OR predict*[tw]) AND (clinical trial*[tw] OR case[tw] OR follow-up*[tw] OR longitud*[tw] OR prospect*[tw] OR retrospect*[tw]) AND 2004:3000[dp]

943

(knee[tw] OR knees[tw]) AND (arthrosis[tw] OR arthrot*[tw] OR degenerative arthrit*[tw] OR degenerative joint disease*[tw] OR osteoarthrit*[tw] OR osteo-arthrit*[tw] OR osteoarthro*[tw] OR osteo-arthro*[tw]) AND (progress*[tw] OR exacerbat*[tw] OR precipit*[tw] OR prognos*[tw] OR predict*[tw]) AND (clinical trial*[tw] OR case[tw] OR follow-up*[tw] OR longitud*[tw] OR prospect*[tw] OR retrospect*[tw])

11-06-30: 1624

11-07-19: 1638

)

| (knee[tw] OR knees[tw]) | | AND | | (arthrosis[tw] OR arthrot*[tw] OR degenerative arthrit*[tw] OR | | | | | | | |
| --- | --- | --- | --- | --- | --- | --- | --- | --- | --- | --- | --- |
| degenerative joint disease*[tw] OR osteoarthrit*[tw] OR osteo-arthrit*[tw] OR osteoarthro*[tw] | | | | | | | | | | | |
| OR osteo-arthro*[tw]) | AND | | (progress*[tw] OR exacerbat*[tw] OR precipit*[tw] | | | | |  | OR | |  |
| prognos*[tw] OR predict*[tw]) | | | | AND | (clinical trial*[tw] OR case[tw] | | OR cases[tw] | | | OR follow- | |
| up*[tw] OR longitud*[tw] OR prospect*[tw] OR retrospect*[tw] | | | | | | OR cohort*[tw] | | |  | | |

11-07-19: 1799

(sharif[1au] OR dieppe[1au] OR zhang[1au] OR sharma[1au] OR bruyere[1au] OR cerejo[1au] OR felson[1au] OR mcalindon[1au] OR spector[1au] OR vilim[1au] OR bagge[1au] OR brandt[1au] OR ledingham[1au] OR miyazaki[1au] OR bettica[1au] OR cooper[1au] OR doherty[1au] OR fraenkel[1au] OR hart[1au] OR lane[1au] OR pavelka[1au] OR schouten[1au] OR sugiyama[1au] OR wolfe[1au]) AND

# EMbase

(((knee OR knees) NEAR/4 (arthrosis OR arthrot* OR arthrit* OR osteoarthrit* OR 'osteo- arthritis' OR osteoarthro* OR 'osteo-arthrosis')) AND (progress* OR exacerbat* OR precipit* OR prognos* OR predict*) AND (clinical trial* OR case OR 'follow-up' OR longitud* OR prospect* OR retrospect*)):ti,ab,de

lim 0-2003 486

lim 2004-2011 1049

(((knee OR knees) NEAR/4 (arthrosis OR arthrot* OR arthrit* OR osteoarthrit* OR 'osteo- arthritis' OR osteoarthro* OR 'osteo-arthrosis')) AND (progress* OR exacerbat* OR precipit* OR prognos* OR predict*) AND (clinical trial* OR case OR 'follow-up' OR longitud* OR prospect* OR retrospect*)):ti,ab,de

all years 1567

11-06-30: 1577

(((knee OR knees) NEAR/4 (arthrosis OR arthrot* OR arthrit* OR osteoarthrit* OR 'osteo- arthritis' OR osteoarthro* OR 'osteo-arthrosis')) AND (progress* OR exacerbat* OR precipit* OR prognos* OR predict*) AND (clinical trial* OR case* OR 'follow-up' OR longitud* OR prospect* OR retrospect* OR cohort*)):ti,ab,de

11-07-19: 1750

(sharif:au OR dieppe:au OR zhang:au OR sharma:au OR bruyere:au OR cerejo:au OR felson:au OR mcalindon:au OR spector:au OR vilim:au OR bagge:au OR brandt:au OR ledingham:au OR miyazaki:au OR bettica:au OR cooper:au OR doherty:au OR fraenkel:au OR hart:au OR lane:au OR pavelka:au OR schouten:au OR sugiyama:au OR wolfe:au)

| Database | numbers | deduplication | total |
| --- | --- | --- | --- |
| PM | 1-1624 | 0 | 1624 |
| EM | 1625-3191 | -875 | 692 |
| total |  | -875 | 2316 |
| June 30 and | July 19 2011: |  |  |

PM 19/7 en EM 19/7 were read deduplicated

| database | Number | dedup | aantal |
| --- | --- | --- | --- |
| PM 30/6/2011 | 1624 | 0 | 1624 |
| EM 30/6/2011 | 1567 | -875 | 692 |
| PM 19/7/2011 | 178 | -7 | 171 |
| EM 19/7/2011 | 124 | -46 | 78 |
| totaal | 3493 | -928 | 2565 |

Total 2565 (1583 published after December 2003 + 982 published before December 2003)

Update search February 19 2013: 354 added, of which 329 imported (25 duplicated).

Total number: 1583 + 329 = 1912 articles.
