# Supplementary material for: What Are the Prognostic Factors for Radiographic Progression of Knee Osteoarthritis? A Meta-analysis
Source: Clin Orthop Relat Res. 2015 May 21;473(9):2969–89. doi: 10.1007/s11999-015-4349-z (PMC4523522; doi:10.1007/s11999-015-4349-z)
Supplement: Supplementary file 2 — Supplementary material 2 (DOC 291 kb) [file 11999_2015_4349_MOESM2_ESM.doc]

**Appendix 2**. Results

Systemic Factors

Three studies found positive associations between age and osteoarthritis (OA) progression . All other authors studying age reported no association with OA progression . Only one study found an association for sex . The remaining eight found no association . Kopec et al. [30] found that blacks were more susceptible to radiographic OA progression compared with whites. Three studies were performed to determine an association for low bone density . Only Zhang et al. [73] found a protective effect of high versus low bone density (fourth, third, and second quartiles versus first all showed associations). Nishimura et al. [46] found no association for osteoporosis. Fraenkel et al. [22] found no association for insulin-like growth factor-1. Schouten et al. [54] found an association only in their third versus first tertile analysis. Yoshimura et al. [70] studied the association with metabolic syndrome (overweight, hypertension, dyslipidemia, impaired glucose tolerance). Having two or more of these components was associated with OA progression. Zhang et al. [74] found no associations for women with past and current estrogen use and women who never used estrogen. Schouten et al. [53] found no association for uric acid concentration. Fayfman et al. [17] found no association for plasma homocysteine levels. Zhai et al. [72] found a genetic influence on the progression of knee OA, mainly in the medial knee compartment, by calculating and comparing hereditary estimates between monozygotic and dizygotic twins. There was no association in the monozygotic but there was in the dizygotic twins. The associations between several single nucleotide polymorphisms and knee OA progression were studied by two groups of authors . Kerna et al. [29] found an association for an rs3740199 polymorphism in women but not in men. An rs1871054 polymorphism was not associated. Valdes et al. [66] found several genes that appeared to influence OA progression. The polymorphisms at ADAM12, CILP, and TNA appeared to correlate; however, only CILP_395 was associated. Wolfe and Lane [69] found no associations for depression or anxiety.

Disease Characteristics

The association for baseline knee pain was studied by numerous groups . Two groups found associations . Multiple studies were performed determining the association for baseline radiographic or clinical OA severity. Bruyere et al. [9] found no association for an initial high radiographic OA score. Duncan et al. [16] reported an association for mild patellofemoral joint OA at baseline. Mazzuca et al. [37] found that a larger joint space width at baseline was associated with a decreased risk of mean change in joint space width, and the presence of patellofemoral OA at baseline was positively associated with change in mean joint space width. They found no association for baseline clinical severity. Ledingham et al. [34] found an association with baseline radiographic OA severity and change in attrition, but not with change in Kellgren-Lawrence score or joint space narrowing. Wolfe and Lane [69] determined an association for an initial high joint space narrowing score and for global severity of symptoms. They found no association with an initial health assessment score. Miyazaki et al. [42] and Pavelka et al. [49] found no association for baseline radiographic severity. Dieppe et al. [14] found no association for baseline clinical knee OA severity. An association for the presence of Heberden nodes was found by Schouten et al. . Cooper et al. [11] and Nishimura et al. [46] found no associations. Haugen et al. [26] found no association for radiographic features of hand OA. Schouten et al. 53] and Ledingham et al. [34] reported positive associations for generalized osteoarthritis. Muraki et al. [43] found no association for hand grip strength. Two studies determined the association between duration of symptoms and OA progression . Only Wolfe and Lane found an association .

Intrinsic Factors

Eight groups of authors studied the association for knee alignment, varus and valgus . Most analyses showed associations; however, Brouwer et al. [7] and Cerejo et al. [10] found that valgus versus neutral-aligned knees had no association. Hunter et al. [27] studied patella alignment on the progression of tibiofemoral OA. They found associations between the bisect offset of the patella and medial and lateral tibiofemoral OA progression. In addition, the patellar tilt was associated with medial tibiofemoral OA progression. Miyazaki et al. [42] found an association in the univariate analysis for varus alignment. Miyazaki et al. [42] found an association for adduction moment. Two studies found no association for former knee injury . Madan-Sharma et al. [36] found no associations for bone marrow lesions; MRI detected subchondral bone cysts, cartilage loss, and joint effusion. They did find an association for meniscal damage detected on MRI. Schouten et al. [53] found no association for meniscectomy or chondrocalcinosis. Felson et al. [18] found an association for tibiofemoral osteophytes. They found a positive association for ipsilateral osteophytes and joint space narrowing and a negative association for contralateral osteophytes. Benichou et al. [3] found no association for osteophytes. Nishimura et al. [46] reported that larger ROM of the knee was associated with less knee OA progression.

Extrinsic Factors

A total of 24 analyses were done determining the association for BMI . Twelve of these 24 analyses found positive associations

. The remaining 12 found no associations. Two studies found no association between quadriceps strength and OA progression .

Golightly et al. [23] found an association comparing leg length inequality with no leg length inequality in patients with baseline Kellgren-Lawrence score of 2 or greater. Harvey et al. [25]

found an increased risk for the shorter leg in patients with a leg length inequality of 1 cm or greater compared with no leg length inequality, but not when comparing leg length inequality of 2 cm or greater compared with no leg length inequality. Miyazaki et al. found that the degree of AP knee laxity was not associated with OA progression; however, the degree of enhanced laxity resulting from exercise was associated with OA progression . Two studies found no association for running on the progression of OA . Cooper et al. [11] found no association for regular sport activities. Schouten et al. [53] analyzed different types of activities: physical activity in general; walking; and squatting/kneeling, but no associations were found. For duration of standing (hours), an association was found in the comparison of the medium duration versus the low duration groups. Two studies determined a protective effect of vitamin D dietary intake . McAlindon et al. [39, 40] also found a protective effect for vitamin D serum levels, vitamin C dietary intake, and β-carotene dietary intake. Felson et al. [20] found no associations for serum vitamin D levels in two cohorts. Peregoy and Wilder [50] found no relation with vitamin C dietary intake. Wilder et al. [68] found a protective role for vitamin intake. Nishimura et al. [46] and Schouten et al. [53] found no associations for smoking.

Markers

The association for baseline serum C-reactive protein levels was reported in three articles . Only Spector et al. [65] reported an association. Attur et al. [1] found that serum levels of IL-1β proved to be a good predictor. Botha-Scheepers et al. [5] however, found no association. Nor did they find an association for IL-1Ra. They did find an association for serum levels of IL-10. Three studies found associations for tumor necrosis factor-α in patients with OA progression . Nelson et al. [44] found no association for serum levels of transforming growth factor-β1. The predictive value of serum levels of hyaluronic acid on OA progression was determined in four studies . All studies reported associations.

Bruyere et al. [8] found an association for serum levels of keratan sulfate. Sharif et al. [55] found no association. Five studies determined the predictive value of serum cartilage oligometric matrix protein levels . Only Sharif et al. [57, 58] and Vilim et al. [67] found an association. Pavelka et al. [48] studied the associations for multiple serum markers, namely serum cartilage oligometric matrix protein, pentosidine, YKL-40, matrix metalloproteinase-9, and tissue inhibitors of metalloproteinase-9. They found an association only for serum pentosidine. Sharif et al. [56] found an association for the serum marker N-propeptide of type II collagen. Two of three studies found associations between urinary crosslinked C-telopeptide and knee OA progression . Larsson et al. [33] found an association for synovial aggrecan neoepitope amino acid sequence levels. Denoble et al. [13] found an association for synovium fluid level of IL-18. Kraus et al. [31] found that fractal signature analysis of the medial tibial plateau was predictive for medial knee joint space narrowing, but not for osteophyte formation or joint space narrowing of the lateral compartment. Mazzuca et al. [38] found no association for 99mTc-MDP uptake on bone scintigraphy.

**References**

1. Attur M, Belitskaya-Levy I, Oh C, Krasnokutsky S, Greenberg J, Samuels J, Smiles S, Lee S, Patel J, Al-Mussawir H, McDaniel G, Kraus VB, Abramson SB. Increased interleukin-1β gene expression in peripheral blood leukocytes is associated with increased pain and predicts risk for progression of symptomatic knee osteoarthritis. *Arthritis Rheum.* 2011;63:1908-1917.

2. Bagge E, Bjelle A, Svanborg A. Radiographic osteoarthritis in the elderly: a cohort comparison and a longitudinal study of the "70-year old people in Goteborg". *Clin Rheumatol.* 1992;11:486-491.

3. Benichou OD, Hunter DJ, Nelson DR, Guermazi A, Eckstein F, Kwoh K, Myers SL, Wirth W, Duryea J; Osteoarthritis Initiative Investigators. One-year change in radiographic joint space width in patients with unilateral joint space narrowing: data from the Osteoarthritis Initiative. *Arthritis Care Res (Hoboken).* 2010;62:924-931.

4. Bergink AP, Uitterlinden AG, Van Leeuwen JP, Buurman CJ, Hofman A, Verhaar JA, Pols HA. Vitamin D status, bone mineral density, and the development of radiographic osteoarthritis of the knee: The Rotterdam Study. *J Clin Rheumatol.* 2009;15:230-237.

5. Botha-Scheepers S, Watt I, Slagboom E, de Craen AJ, Meulenbelt I, Rosendaal FR, Breedveld FC, Huizinga TW, Kloppenburg M. Innate production of tumour necrosis factor alpha and interleukin 10 is associated with radiological progression of knee osteoarthritis. *Ann Rheum Dis.* 2008;67:1165-1169.

6. Brandt KD, Heilman DK, Slemenda C, Katz BP, Mazzuca SA, Braunstein EM, Byrd D. Quadriceps strength in women with radiographically progressive osteoarthritis of the knee and those with stable radiographic changes. *J Rheumatol.* 1999;26:2431-2437.

7. Brouwer GM, van Tol AW, Bergink AP, Belo JN, Bernsen RM, Reijman M, Pols HA, Bierma-Zeinstra SM. Association between valgus and varus alignment and the development and progression of radiographic osteoarthritis of the knee. *Arthritis Rheum.* 2007;56:1204-1211.

8. Bruyere O, Collette JH, Ethgen O, Rovati LC, Giacovelli G, Henrotin YE, Seidel L, Reginster JY. Biochemical markers of bone and cartilage remodeling in prediction of longterm progression of knee osteoarthritis. *J Rheumatol.* 2003;30:1043-1050.

9. Bruyere O, Honore A, Ethgen O, Rovati LC, Giacovelli G, Henrotin YE, Seidel L, Reginster JY. Correlation between radiographic severity of knee osteoarthritis and future disease progression: results from a 3-year prospective, placebo-controlled study evaluating the effect of glucosamine sulfate. *Osteoarthritis Cartilage.* 2003;11:1-5.

10. Cerejo R, Dunlop DD, Cahue S, Channin D, Song J, Sharma L. The influence of alignment on risk of knee osteoarthritis progression according to baseline stage of disease. *Arthritis Rheum.* 2002;46:2632-2636.

11. Cooper C, Snow S, McAlindon TE, Kellingray S, Stuart B, Coggon D, Dieppe PA. Risk factors for the incidence and progression of radiographic knee osteoarthritis. *Arthritis Rheum.* 2000;43:995-1000.

12. Dam EB, Byrjalsen I, Karsdal MA, Qvist P, Christiansen C. Increased urinary excretion of C-telopeptides of type II collagen (CTX-II) predicts cartilage loss over 21 months by MRI. *Osteoarthritis Cartilage.* 2009;17:384-389.

13. Denoble AE, Huffman KM, Stabler TV, Kelly SJ, Hershfield MS, McDaniel GE, Coleman RE, Kraus VB. Uric acid is a danger signal of increasing risk for osteoarthritis through inflammasome activation. *Proc Natl Acad Sci U S A.* 2011;108:2088-2093.

14. Dieppe P, Cushnaghan J, Shepstone L. The Bristol 'OA500' study: progression of osteoarthritis (OA) over 3 years and the relationship between clinical and radiographic changes at the knee joint. *Osteoarthritis Cartilage.* 1997;5:87-97.

15. Dieppe P, Cushnaghan J, Young P, Kirwan J. Prediction of the progression of joint space narrowing in osteoarthritis of the knee by bone scintigraphy. *Ann Rheum Dis.* 1993;52:557-563.

16. Duncan R, Peat G, Thomas E, Hay EM, Croft P. Incidence, progression and sequence of development of radiographic knee osteoarthritis in a symptomatic population. *Ann Rheum Dis.* 2011;70:1944-1948.

17. Fayfman M, Niu J, Zhang YQ, Felson DT, Sack B, Aliabadi P, Selhub J, Hunter DJ. The relation of plasma homocysteine to radiographic knee osteoarthritis. *Osteoarthritis Cartilage.* 2009;17:766-771.

18. Felson DT, Gale DR, Elon Gale M, Niu J, Hunter DJ, Goggins J, Lavalley MP. Osteophytes and progression of knee osteoarthritis. *Rheumatology (Oxford).* 2005;44:100-104.

19. Felson DT, Goggins J, Niu J, Zhang Y, Hunter DJ. The effect of body weight on progression of knee osteoarthritis is dependent on alignment. *Arthritis Rheum.* 2004;50:3904-3909.

20. Felson DT, Niu J, Clancy M, Aliabadi P, Sack B, Guermazi A, Hunter DJ, Amin S, Rogers G, Booth SL. Low levels of vitamin D and worsening of knee osteoarthritis: results of two longitudinal studies. *Arthritis Rheum.* 2007;56:129-136.

21. Felson DT, Zhang Y, Hannan MT, Naimark A, Weissman BN, Aliabadi P, Levy D. The incidence and natural history of knee osteoarthritis in the elderly: The Framingham Osteoarthritis Study. *Arthritis Rheum.* 1995;38:1500-1505.

22. Fraenkel L, Zhang Y, Trippel SB, McAlindon TE, LaValley MP, Assif A, Adams KE, Evans SR, Felson DT. Longitudinal analysis of the relationship between serum insulin-like growth factor-I and radiographic knee osteoarthritis. *Osteoarthritis Cartilage.* 1998;6:362-367.

23. Golightly YM, Allen KD, Helmick CG, Schwartz TA, Renner JB, Jordan JM. Hazard of incident and progressive knee and hip radiographic osteoarthritis and chronic joint symptoms in individuals with and without limb length inequality. *J Rheumatol.* 2010;37:2133-2140.

24. Hart DJ, Cronin C, Daniels M, Worthy T, Doyle DV, Spector TD. The relationship of bone density and fracture to incident and progressive radiographic osteoarthritis of the knee: the Chingford Study. *Arthritis Rheum.* 2002;46:92-99.

25. Harvey WF, Yang M, Cooke TD, Segal NA, Lane N, Lewis CE, Felson DT. Association of leg-length inequality with knee osteoarthritis: a cohort study. *Ann Intern Med.* 2010;152:287-295.

26. Haugen IK, Cotofana S, Englund M, Kvien TK, Dreher D, Nevitt M, Lane NE, Eckstein F; Osteoarthritis Initiative Investigators. Hand joint space narrowing and osteophytes are associated with magnetic resonance imaging-defined knee cartilage thickness and radiographic knee osteoarthritis: data from the Osteoarthritis Initiative. *J Rheumatol.* 2012;39:161-166.

27. Hunter DJ, Zhang YQ, Niu JB, Felson DT, Kwoh K, Newman A, Kritchevsky S, Harris T, Carbone L, Nevitt M. Patella malalignment, pain and patellofemoral progression: the Health ABC Study. *Osteoarthritis Cartilage.* 2007;15:1120-1127.

28. Kerkhof HJ, Bierma-Zeinstra SM, Castano-Betancourt MC, de Maat MP, Hofman A, Pols HA, Rivadeneira F, Witteman JC, Uitterlinden AG, van Meurs JB. Serum C reactive protein levels and genetic variation in the CRP gene are not associated with the prevalence, incidence or progression of osteoarthritis independent of body mass index. *Ann Rheum Dis.* 2010;69:1976-1982.

29. Kerna I, Kisand K, Tamm AE, Lintrop M, Veske K, Tamm AO. Missense single nucleotide polymorphism of the ADAM12 gene is associated with radiographic knee osteoarthritis in middle-aged Estonian cohort. *Osteoarthritis Cartilage.* 2009;17:1093-1098.

30. Kopec JA, Sayre EC, Schwartz TA, Renner JB, Helmick CG, Badley EM, Cibere J, Callahan LF, Jordan JM. Occurrence of radiographic osteoarthritis of the knee and hip among African Americans and whites: a population-based prospective cohort study. *Arthritis Care Res (Hoboken).* 2013;65:928-935.

31. Kraus VB, Feng S, Wang S, White S, Ainslie M, Brett A, Holmes A, Charles HC. Trabecular morphometry by fractal signature analysis is a novel marker of osteoarthritis progression. *Arthritis Rheum.* 2009;60:3711-3722.

32. Lane NE, Oehlert JW, Bloch DA, Fries JF. The relationship of running to osteoarthritis of the knee and hip and bone mineral density of the lumbar spine: a 9 year longitudinal study. *J Rheumatol.* 1998;25:334-341.

33. Larsson S, Englund M, Struglics A, Lohmander LS. The association between changes in synovial fluid levels of ARGS-aggrecan fragments, progression of radiographic osteoarthritis and self-reported outcomes: a cohort study. *Osteoarthritis Cartilage.* 2012;20:388-395.

34. Ledingham J, Regan M, Jones A, Doherty M. Factors affecting radiographic progression of knee osteoarthritis. *Ann Rheum Dis.* 1995;54:53-58.

35. Le Graverand MP, Brandt K, Mazzuca SA, Raunig D, Vignon E. Progressive increase in body mass index is not associated with a progressive increase in joint space narrowing in obese women with osteoarthritis of the knee. *Ann Rheum Dis.* 2009;68:1734-1738.

36. Madan-Sharma R, Kloppenburg M, Kornaat PR, Botha-Scheepers SA, Le Graverand MP, Bloem JL, Watt I. Do MRI features at baseline predict radiographic joint space narrowing in the medial compartment of the osteoarthritic knee 2 years later? *Skeletal Radiol.* 2008;37:805-811.

37. Mazzuca SA, Brandt KD, Katz BP, Ding Y, Lane KA, Buckwalter KA. Risk factors for progression of tibiofemoral osteoarthritis: an analysis based on fluoroscopically standardised knee radiography. *Ann Rheum Dis.* 2006;65:515-519.

38. Mazzuca SA, Brandt KD, Schauwecker DS, Buckwalter KA, Katz BP, Meyer JM, Lane KA. Bone scintigraphy is not a better predictor of progression of knee osteoarthritis than Kellgren and Lawrence grade. *J Rheumatol.* 2004;31:329-332.

39. McAlindon TE, Felson DT, Zhang Y, Hannan MT, Aliabadi P, Weissman B, Rush D, Wilson PW, Jacques P. Relation of dietary intake and serum levels of vitamin D to progression of osteoarthritis of the knee among participants in the Framingham Study. *Ann Intern Med.* 1996;125:353-359.

40. McAlindon TE, Jacques P, Zhang Y, Hannan MT, Aliabadi P, Weissman B, Rush D, Levy D, Felson DT. Do antioxidant micronutrients protect against the development and progression of knee osteoarthritis? *Arthritis Rheum.* 1996;39:648-656.

41. Miyazaki T, Uchida K, Sato M, Watanabe S, Yoshida A, Wada M, Shimada S, Kuiper JH, Baba H. Knee laxity after staircase exercise predicts radiographic disease progression in medial compartment knee osteoarthritis. *Arthritis Rheum.* 2012;64:3908-3916.

42. Miyazaki T, Wada M, Kawahara H, Sato M, Baba H, Shimada S. Dynamic load at baseline can predict radiographic disease progression in medial compartment knee osteoarthritis. *Ann Rheum Dis.* 2002;61:617-622.

43. Muraki S, Akune T, Oka H, Ishimoto Y, Nagata K, Yoshida M, Tokimura F, Nakamura K, Kawaguchi H, Yoshimura N. Incidence and risk factors for radiographic knee osteoarthritis and knee pain in Japanese men and women: a longitudinal population-based cohort study. *Arthritis Rheum.* 2012;64:1447-1456.

44. Nelson AE, Golightly YM, Kraus VB, Stabler T, Renner JB, Helmick CG, Jordan JM. Serum transforming growth factor-beta 1 is not a robust biomarker of incident and progressive radiographic osteoarthritis at the hip and knee: the Johnston County Osteoarthritis Project. *Osteoarthritis Cartilage.* 2010;18:825-829.

45. Nevitt MC, Zhang Y, Javaid MK, Neogi T, Curtis JR, Niu J, McCulloch CE, Segal NA, Felson DT. High systemic bone mineral density increases the risk of incident knee OA and joint space narrowing, but not radiographic progression of existing knee OA: the MOST study. *Ann Rheum Dis.* 2010;69:163-168.

46. Nishimura A, Hasegawa M, Kato K, Yamada T, Uchida A, Sudo A. Risk factors for the incidence and progression of radiographic osteoarthritis of the knee among Japanese. *Int Orthop.* 2011;35:839-843

47. Niu J, Zhang YQ, Torner J, Nevitt M, Lewis CE, Aliabadi P, Sack B, Clancy M, Sharma L, Felson DT. Is obesity a risk factor for progressive radiographic knee osteoarthritis? *Arthritis Rheum.* 2009;61:329-335.

48. Pavelka K, Forejtova S, Olejarova M, Gatterova J, Senolt L, Spacek P, Braun M, Hulejova M, Stovickova J, Pavelkova A. Hyaluronic acid levels may have predictive value for the progression of knee osteoarthritis. *Osteoarthritis Cartilage.* 2004;12:277-283.

49. Pavelka K, Gatterova J, Altman RD. Radiographic progression of knee osteoarthritis in a Czech cohort. *Clin Exp Rheumatol.* 2000;18:473-477.

50. Peregoy J, Wilder FV. The effects of vitamin C supplementation on incident and progressive knee osteoarthritis: a longitudinal study. *Public Health Nutr.* 2011;14:709-715.

51. Reijman M, Hazes JM, Bierma-Zeinstra SM, Koes BW, Christgau S, Christiansen C, Uitterlinden AG, Pols HA. A new marker for osteoarthritis: cross-sectional and longitudinal approach. *Arthritis Rheum.* 2004;50:2471-2478.

52. Reijman M, Pols HA, Bergink AP, Hazes JM, Belo JN, Lievense AM, Bierma-Zeinstra SM. Body mass index associated with onset and progression of osteoarthritis of the knee but not of the hip: the Rotterdam Study. *Ann Rheum Dis.* 2007;66:158-162.

53. Schouten JS, van den Ouweland FA, Valkenburg HA. A 12 year follow up study in the general population on prognostic factors of cartilage loss in osteoarthritis of the knee. *Ann Rheum Dis.* 1992;51:932-937.

54. Schouten JS, Van den Ouweland FA, Valkenburg HA, Lamberts SW. Insulin-like growth factor-1: a prognostic factor of knee osteoarthritis. *Br J Rheumatol.* 1993;32:274-280.

55. Sharif M, George E, Shepstone L, Knudson W, Thonar EJ, Cushnaghan J, Dieppe P. Serum hyaluronic acid level as a predictor of disease progression in osteoarthritis of the knee. *Arthritis Rheum.* 1995;38:760-767.

56. Sharif M, Kirwan J, Charni N, Sandell LJ, Whittles C, Garnero P. A 5-yr longitudinal study of type IIA collagen synthesis and total type II collagen degradation in patients with knee osteoarthritis: association with disease progression. *Rheumatology (Oxford).* 2007;46:938-943.

57. Sharif M, Kirwan JR, Elson CJ, Granell R, Clarke S. Suggestion of nonlinear or phasic progression of knee osteoarthritis based on measurements of serum cartilage oligomeric matrix protein levels over five years. *Arthritis Rheum.* 2004;50:2479-2488.

58. Sharif M, Saxne T, Shepstone L, Kirwan JR, Elson CJ, Heinegard D, Dieppe PA. Relationship between serum cartilage oligomeric matrix protein levels and disease progression in osteoarthritis of the knee joint. *Br J Rheumatol.* 1995;34:306-310.

59. Sharif M, Shepstone L, Elson CJ, Dieppe PA, Kirwan JR. Increased serum C reactive protein may reflect events that precede radiographic progression in osteoarthritis of the knee. *Ann Rheum Dis.* 2000;59:71-74.

60. Sharma L, Dunlop DD, Cahue S, Song J, Hayes KW. Quadriceps strength and osteoarthritis progression in malaligned and lax knees. *Ann Intern Med.* 2003;138:613-619.

61. Sharma L, Song J, Dunlop D, Felson D, Lewis CE, Segal N, Torner J, Cooke TD, Hietpas J, Lynch J, Nevitt M. Varus and valgus alignment and incident and progressive knee osteoarthritis. *Ann Rheum Dis.* 2010;69:1940-1945.

62. Sharma L, Song J, Felson DT, Cahue S, Shamiyeh E, Dunlop DD. The role of knee alignment in disease progression and functional decline in knee osteoarthritis. *JAMA.* 2001;286:188-195.

63. Spector TD, Dacre JE, Harris PA, Huskisson EC. Radiological progression of osteoarthritis: an 11 year follow up study of the knee. *Ann Rheum Dis.* 1992;51:1107-1110.

64. Spector TD, Hart DJ, Doyle DV. Incidence and progression of osteoarthritis in women with unilateral knee disease in the general population: the effect of obesity. *Ann Rheum Dis.* 1994;53:565-568.

65. Spector TD, Hart DJ, Nandra D, Doyle DV, Mackillop N, Gallimore JR, Pepys MB. Low-level increases in serum C-reactive protein are present in early osteoarthritis of the knee and predict progressive disease. *Arthritis Rheum.* 1997;40:723-727.

66. Valdes AM, Hart DJ, Jones KA, Surdulescu G, Swarbrick P, Doyle DV, Schafer AJ, Spector TD. Association study of candidate genes for the prevalence and progression of knee osteoarthritis. *Arthritis Rheum.* 2004;50:2497-2507.

67. Vilim V, Olejarova M, Machacek S, Gatterova J, Kraus VB, Pavelka K. Serum levels of cartilage oligomeric matrix protein (COMP) correlate with radiographic progression of knee osteoarthritis. *Osteoarthritis Cartilage.* 2002;10:707-713.

68. Wilder FV, Leaverton PE, Rogers MW, Lemrow NB. Vitamin supplements and radiographic knee osteoarthritis: The Clearwater Osteoarthritis Study. *J Musculoskelet Res.* 2009;12:85-93.

69. Wolfe F, Lane NE. The longterm outcome of osteoarthritis: rates and predictors of joint space narrowing in symptomatic patients with knee osteoarthritis. *J Rheumatol.* 2002;29:139-146.

70. Yoshimura N, Muraki S, Oka H, Tanaka S, Kawaguchi H, Nakamura K, Akune T. Accumulation of metabolic risk factors such as overweight, hypertension, dyslipidaemia, and impaired glucose tolerance raises the risk of occurrence and progression of knee osteoarthritis: a 3-year follow-up of the ROAD study. *Osteoarthritis Cartilage.* 2012;20:1217-1226.

71. Yusuf E, Bijsterbosch J, Slagboom PE, Rosendaal FR, Huizinga TW, Kloppenburg M. Body mass index and alignment and their interaction as risk factors for progression of knees with radiographic signs of osteoarthritis. *Osteoarthritis Cartilage.* 2011;19:1117-1122.

72. Zhai G, Hart DJ, Kato BS, MacGregor A, Spector TD. Genetic influence on the progression of radiographic knee osteoarthritis: a longitudinal twin study. *Osteoarthritis Cartilage.* 2007;15:222-225.

73. Zhang Y, Hannan MT, Chaisson CE, McAlindon TE, Evans SR, Aliabadi P, Levy D, Felson DT. Bone mineral density and risk of incident and progressive radiographic knee osteoarthritis in women: the Framingham Study. *J Rheumatol.* 2000;27:1032-1037.

74. Zhang Y, McAlindon TE, Hannan MT, Chaisson CE, Klein R, Wilson PW, Felson DT. Estrogen replacement therapy and worsening of radiographic knee osteoarthritis: the Framingham Study. *Arthritis Rheum.* 1998;41:1867-1873.
